# Immediate neural effects of acupuncture manipulation time for stroke with motor dysfunction: a fMRI pilot study

**DOI:** 10.3389/fnins.2023.1297149

**Published:** 2024-01-05

**Authors:** Yihao Zhou, Anhong Dai, Sifeng Feng, Tao Zhu, Meifang Liu, Jing Shi, Dongyan Wang

**Affiliations:** ^1^Heilongjiang University of Chinese Medicine, Harbin, China; ^2^The First Affiliated Hospital of Yunnan University of Chinese Medicine, Yunnan Provincial Hospital of Traditional Chinese Medicine, Kunming, China; ^3^Yan’an Hospital Affiliated to Kunming Medical University, Kunming, China; ^4^Second Affiliated Hospital, Heilongjiang University of Chinese Medicine, Harbin, China

**Keywords:** stroke, motor dysfunction, acupuncture manipulation time, neural effects, regional homogeneity, pilot study

## Abstract

**Introduction:**

Acupuncture is widely utilized as a beneficial intervention for the treatment of motor dysfunction after stroke, and its effectiveness depends on the stimulation dose. Manipulation time is an important factor affecting the dose. This trial aimed use fMRI to explore the immediate neural effects in stroke patients with motor dysfunction by different acupuncture manipulation times, to reveal the neural mechanism of acupuncture manipulation.

**Methods:**

Thirty participants were divided into three groups according to different acupuncture times. Each group received the same acupoint prescription, although the continuous manipulation time of each acupoint in three groups was 1-min, 2-min, and 3-min, respectively. The NIHSS, FMA and fMRI-BOLD in each participant we obtained before and after acupuncture manipulation. Then, we used the regional homogeneity (ReHo) algorithm to analyze the changes of brain function and to compare the neural effects at different acupuncture manipulation times.

**Results:**

There were no significant differences in NIHSS and FMA scores between and within groups. Longitudinal analysis of ReHo values indicated that the right inferior frontal gyrus was activated in the 1-min group, the right insula in the 2-min group, and the right inferior temporal gyrus in the 3-min group. Compared with the 1-min group, the 2-min group showed the ReHo values of the right precentral gyrus was decreased, and the 3-min group showed the left cerebellum posterior lobe was increased, the right posterior cingulate gyrus and the right anterior cingulate gyrus were decreased. Compared with the 2-min group, the 3-min group showed the ReHo values of the right cerebellum anterior lobe was increased.

**Conclusion:**

Our findings suggest that acupuncture at different manipulation times caused different changes of the neural effects in stroke patients, and the volume of activated voxel clusters is positively correlated with the manipulation time. Longer acupuncture manipulation could drive SMN and DMN in stroke patients, which may be the potential neurological mechanism of acupuncture manipulation affecting the recovery of motor dysfunction.

## Introduction

According to the World Health Organization (WHO) in 2019, stroke is the second leading cause of death (Feigin et al., 2022). In China, stroke has become a major chronic noninfectious illness that seriously endangers the health of Chinese people due to the aging of the population and the unequal distribution of medical resources (Zhou et al., 2016; Wu et al., 2019). Additionally, stroke is one of the leading causes of disability worldwide (Badawi et al., 2023). Motor dysfunction affects more than half of stroke patients and has a significant detrimental influence on their daily lives (Lee et al., 2015).

Acupuncture is recommended by the WHO as a complementary therapy for post-stroke rehabilitation (Chavez et al., 2017). Research has shown that acupuncture can enhance muscle strength, muscle tone, and balance function in stroke patients (Fan et al., 2020; Guo and Cheng, 2022; Zhang et al., 2022). Therefore, it is widely utilized as a beneficial intervention to improve motor dysfunction. Generally, the effectiveness of acupuncture depends on the stimulation does. Acupuncture manipulation time is an important factor affecting the dose, but acupuncturists often disregard this issue in practice. This was also confirmed by our previous investigation, which found that there was no uniform standard of acupuncture manipulation time in various studies (Zhou et al., 2022). In the research of acupuncture for stroke, more studies used 1-min manipulation time, but also involved other manipulation time such as 2 min, 3 min, etc. What effects might different acupuncture manipulation times have on stroke patients with motor dysfunction? The answer to this question remains unclear but deserves further clarity.

Research has confirmed that neuroimaging technologies may precisely reflect the characteristics of brain function in neuropsychiatric disorders and therapeutic responses (Dan, 2019; Risacher and Saykin, 2021). To better understand the underlying mechanism of acupuncture, an increasing number of acupuncture research have started to explore the brain neural effects linked to therapeutic responses applied functional magnetic resonance imaging (fMRI) techniques (Cai et al., 2018; Zhang et al., 2021a). Neuroimaging studies have tentatively confirmed that acupuncture could promote neuroplasticity in cortical motor areas in stroke patients, which improves motor disorders (Lv et al., 2021; Zhang et al., 2021a).

Regional homogeneity (ReHo) is a classical algorithm used to explain variations in brain functional properties, which is mainly employed to assess the temporal homogeneity of adjacent voxels in the active brain area (Zang et al., 2004; Zhao et al., 2017). ReHo believes that when a functional area of the brain is involved in a specific condition, voxels in that area are more uniform in time. Our study focuses on the neural effect of acupuncture manipulation time, and ReHo algorithm can better explain the synchronous activity of adjacent voxels at the same time (Lv et al., 2018).

Here, we used fMRI to investigate the immediate neural effects of different acupuncture manipulation times for stroke patients with motor dysfunction, to explore whether neuroplasticity of motor function after stroke is related to acupuncture manipulation time, and to reveal the potential neural mechanism of acupuncture manipulation.

## Materials and methods

### Participants

This was a pilot study, we decided to enroll 10 subjects per group for a total sample size of 30 based on the minimum requirements of the fMRI study (Desmond and Glover, 2002). From October 2020 to December 2021, 30 stroke patients with motor dysfunction were enrolled at the First Affiliated Hospital of Yunnan University of Chinese Medicine, Affiliated Hospital of Yunnan University, and Yan’an Hospital Affiliated to Kunming Medical University. The First Affiliated Hospital of Yunnan University of Chinese Medicine’s ethics committee authorized this study protocol, which has been registered in the Chinese Clinical Trial Register with the number ChiCTR1900023169.^1^ Each participant was informed of the possible risks and benefits of this study and signed an informed consent form prior to participation.

Patients were enrolled if they met the following inclusion criteria: (1) ischemic stroke diagnoses according to the International Classification of Diseases, 10th reversion (ICD-10-I63.902)^2^; (2) motor dysfunction; (3) first onset and the disease course less than 3 months; and (4) age between 40 and 80 years old, right-handed. Patients were excluded if they (1) had participated in other clinical trials within the last 2 weeks; (2) had serious underlying diseases or complications; (3) women who were pregnant, menstruating, lactating; and (4) had contraindications of MRI scan.

The central randomization system randomly assigned 30 participants to three groups according to different acupuncture times. Based on a special six-digit random number that was automatically produced by the system, each participant was assigned to any group. The group assignment was concealed from participants.

### Treatment protocol

#### Acupuncture treatment

Each group received the same acupoint prescription, which included *Dazhui* (GV14), *Zhiyang* (GV9) and *Mingmen* (GV4). The location and operation of acupoints adhered to Nomenclature and Location of Meridian Points, the National Standard of the People’s Republic of China in 2021.^3^ Three acupoints were manipulated one by one in sequence after the needle produced the *Deqi* sensation (a soreness, numbness, and heaviness sensation). Each acupoint in the three groups received continuous manipulation for 1-min, 2-min, and 3-min, respectively. The acupuncturist held the needle handle with the thumb and forefinger and rotated the needle body in an alternating clockwise and counterclockwise manner, the twist frequency was 120 times per minute and the angle was about 180°. The needles had been pulled out immediately after the manipulation was completed. All participants received only one acupuncture session, which was performed by an attending acupuncturist named Sifeng Feng.

#### Conventional treatment

Conventional therapies that complied with clinical treatment guidelines were allowed during the trial, including different forms of physical rehabilitation and secondary prevention of cerebral infarction. The details of these conventional treatments were documented in detail.

### Outcome measurement

The National Institute of Health Stroke Scale (NIHSS) (Kwah and Diong, 2014) and the Fugl-Meyer motor function assessment (FMA) (Sullivan et al., 2011) were used to assess all participants’ clinical outcomes both before and after acupuncture manipulation. The NIHSS score was used to evaluate the degree of stroke-related functional impairment. It comprises 11 items, and the score, which ranges from 0 to 42, is positively connected with the severity of the impairment. The FMA score was used to evaluate motor dysfunction in stroke patients. A total of 50 items were assessed for the upper and lower limbs, and each symptom received a score between 0 to 2. The better function was indicated by higher scores. Mild motor dysfunction was indicated by a score of 100 to 96, moderate motor dysfunction by a score of 95 to 85, significant motor dysfunction by a score of 85 to 50, and severe motor dysfunction by a score of less than 50. The outcome measurement was completed by an independent evaluator who did not clear what manipulation each participant received.

### Functional MRI scan

All subjects received MRI scans before and after the acupuncture manipulation. MRI data were collected at the imaging department of the Affiliated Hospital of Yunnan University with a 3.0 T magnetic resonance scanner (Siemens Medical, Germany). Participants should keep their eyes closed the entire scanning procedure, but not allowed to meditate or fall asleep. The scanning procedure takes about 10 min and included localization imaging, high-resolution three-dimensional T1-weighted imaging (3D-T1WI), and fMRI blood oxygenation level dependent imaging (fMRI-BOLD).

Magnetic resonance scanning parameters: 3D-T1WI: TR = 1900 ms, TE = 2.26 ms, FOV = 256 mm × 256 mm, matrix = 256 × 256, gap = 0 mm. fMRI-BOLD: TR = 2000 ms, TE = 30 ms, FOV = 240 mm × 240 mm, matrix = 64 × 64, flip angle = 90°, slice thickness = 3 mm. Continuous uninterrupted scanning and covering the entire brain.

### Statistical analysis

#### Clinical data analysis

Clinical data mainly included demographic data and efficacy assessment, which analysis was performed by SPSS 21.0 (IBM, Chicago, IL, United States). Pearson Chi-square was used to investigate gender differences between groups at baseline. Other baseline data such as age, height and weight were analyzed by one-way ANOVA. A paired *T*-test was applied to compare the within-group differences of NHISS and FMA scores, and an analysis of variance was applied for between-group differences. The results were statistically significant at *p* < 0.05.

#### ReHo analysis

The fMRI data analysis was conducted by Data Processing and Analysis for Brain Imaging (DPABI) (Yan et al., 2016) based on MATLAB 2015a platform (MathWorks, Inc., Natick, MA, United States). The steps of imaging data pre-processing were as follows: (1) format conversion and classification of the original data obtained by fMRI scanning; (2) discarded of the first five time points of each subject’s data to reduce signal interference; (3) time level correction; (4) eliminated the data whose head movement was greater than 2.5 mm or rotating movement was greater than 2°; (5) conducted spatial standardization of all corrected data, the standard spatial template provided by Montreal Neurological Institute (MNI) was adopted in our study, and voxels were re-sampled to 3-mm^3^; (6) detrended and filtered with 0.01–0.08 Hz.

The Data Processing Assistant for Resting-State fMRI (DPARSF) was used to perform ReHo analysis after pre-processing. The first step was to calculate the Kendall’s Coefficient of Concordance (KCC) between each voxel and its neighboring voxel. The standardized ReHo value was then calculated to create the ReHo graph by dividing the KCC value of each voxel by the average KCC value of the entire brain. Finally, 8-mm^3^ half-height and full-width Gaussian kernel was used for spatial smoothing to further eliminate noise and increase comparability. A paired *T*-test was performed to compare within-group in ReHo values differences. ANOVA was used for comparison between groups, and when the results showed significance, the difference between-group was compared by the two-sample *T*-test of the two groups to reveal the difference neural effects between the two groups. False discovery rate (FDR) correction was applied to the ReHo results, with a level of *p* < 0.05.

## Results

### Demographics and clinical information

In all, 30 stroke patients with motor dysfunction participated in this study. All participants completed the trial procedures in accordance with the technical route of originally designed. Table 1 demonstrates that at baseline, there were no statistically significant differences in gender, age, height, or weight between the three groups (*p >* 0.05).

**Table 1:** Demographics information of three groups at baseline.

Demographics	1-min group (*n* = 10)	2-min group (*n* = 10)	3-min group (*n* = 10)	*p*-value
Gender (M/F)	6/4	6/4	7/3	0.866
Age (years)	61.00±9.63	57.50±10.71	63.80±10.60	0.405
Height (cm)	164.90±8.84	166.50±7.81	167.80±7.15	0.720
Weight (kg)	63.50±7.95	62.40±10.17	65.60±8.37	0.718

Values are presented as the mean ± SD.

Meanwhile, the NIHSS and FMA scores were not statistically significant between-group differences at baseline (*p >* 0.05). We continued to perform longitudinal comparisons, but unfortunately, the NIHSS and FMA scores of each group were not significantly improved after acupuncture manipulation (*p >* 0.05, Table 2).

**Table 2:** Longitudinal comparison of clinical outcomes in three groups.

Group	NIHSS scores			FMA scores		
	Before	After	*p*-value	Before	After	*p*-value
1-min group (*n* = 10)	4.40±2.12	4.30±2.00	0.343	92.50±3.24	92.80±2.90	0.081
2-min group (*n* = 10)	4.60±3.17	4.50±3.21	0.343	92.30±4.52	92.70±4.62	0.104
3-min group (*n* = 10)	4.40±1.96	4.10±1.66	0.081	92.80±2.57	93.00±2.63	0.168

Values are presented as the mean ± SD.

### ReHo analysis results

#### The difference in ReHo values within three groups

**Figure 1:**
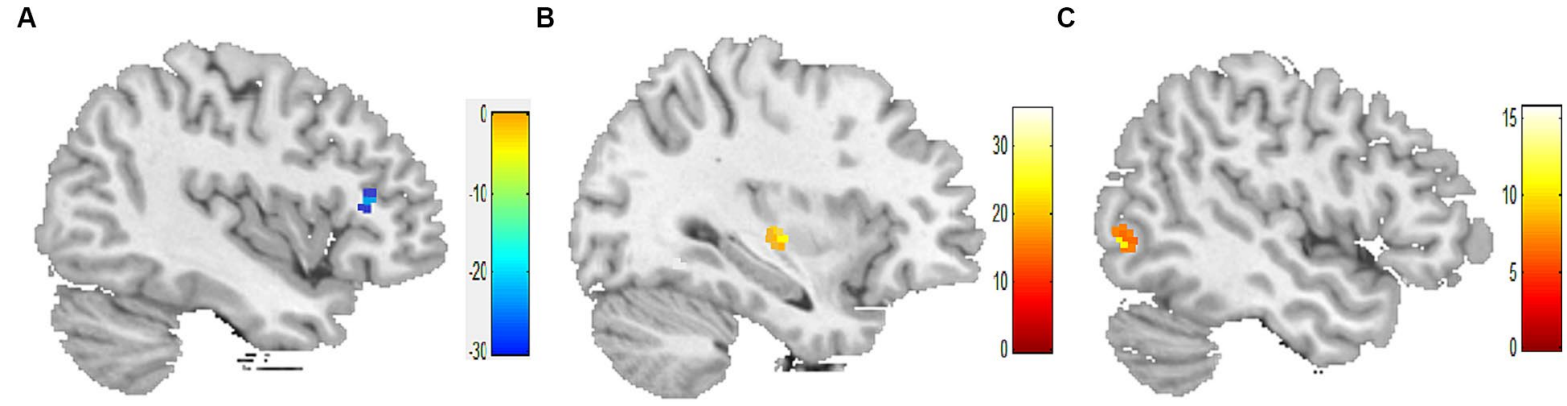
Within-group differences in ReHo values. **(A)** ReHo values was decreased in the right inferior frontal gyrus after 1-min acupuncture manipulation. **(B)** ReHo values was increased in the right insula after 2-min acupuncture manipulation. **(C)** ReHo values was increased in the right inferior temporal gyrus after 3-min acupuncture manipulation. *p* < 0.05, FDR corrected, voxels > 15.

In comparison to before the acupuncture manipulation, the ReHo values of the right inferior frontal gyrus decreased in the 1-min group (Figure 1A), the right insula increased in the 2-min group (Figure 1B), and the right inferior temporal gyrus increased in the 3-min group (Figure 1C). The volume of activated voxel clusters was the largest in the 3-min group and the smallest in the 1-min group. Table 3 provides further information.

**Table 3:** Longitudinal comparison of ReHo values in three groups.

Group	Cluster regions	R/L	Voxels	MNI			*T*-value
				*X*	*Y*	*Z*	
1-min group (*n* = 10)	Inferior frontal gyrus	R	16	45	30	9	−29.98
2-min group (*n* = 10)	Insula	R	24	39	−15	6	35.41
3-min group (*n* = 10)	Inferior temporal gyrus	R	46	51	−69	−3	11.52

#### The difference in ReHo values between three groups

**Figure 2:**
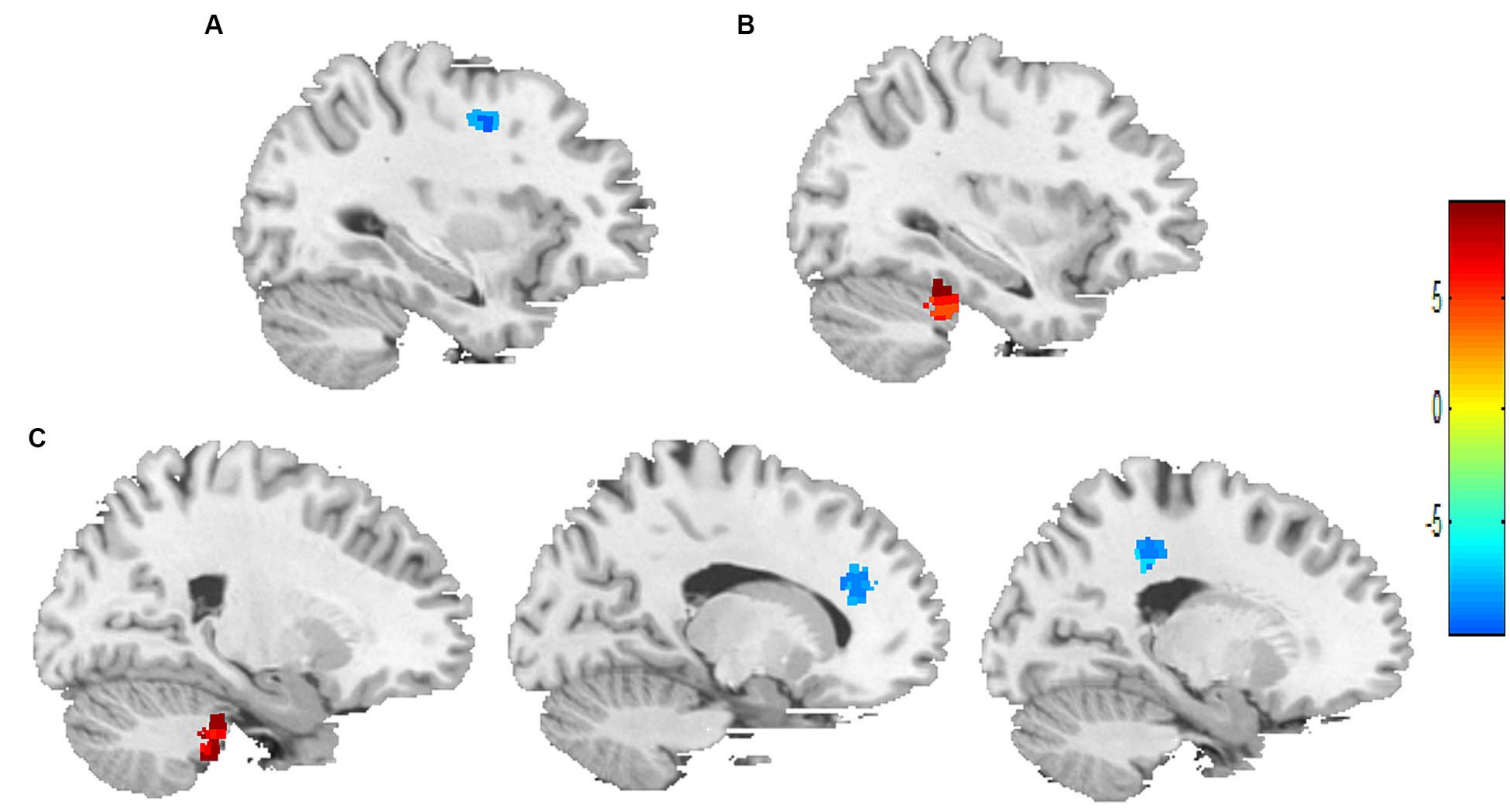
Between-group differences in ReHo values after acupuncture manipulation. **(A)** 2-min acupuncture manipulation decreased ReHo values in the right precentral gyrus compared to 1-min. **(B)** 3-min acupuncture manipulation increased ReHo values in the right cerebellum anterior lobe compared to 2-min. **(C)** Compared with 1-min, 3-min acupuncture manipulation affected ReHo values in the left cerebellum posterior lobe, right posterior cingulate gyrus and right anterior cingulate gyrus. *p* < 0.05, FDR corrected, voxels > 15.

Compared with the 1-min group, the 2-min group showed the ReHo values of the right precentral gyrus was decreased (Figure 2A), and the 3-min group showed the ReHo values of the left cerebellum posterior lobe was increased, the right posterior cingulate gyrus and the right anterior cingulate gyrus were decreased (Figure 2C). Compared with the 2-min group, the 3-min group showed the ReHo values of the right cerebellum anterior lobe was increased (Figure 2B). Table 4 provides further information.

**Table 4:** The difference in ReHo values between three groups after acupuncture manipulation.

Conditions	Cluster regions	R/L	Voxels	MNI			*T*-value
				*X*	*Y*	*Z*	
2-min *>* 1-min	Precentral gyrus	R	18	33	0	42	−7.77
3-min *>* 2-min	Cerebellum anterior lobe	R	27	33	−36	−30	6.8
3-min *>* 1-min	Cerebellum posterior lobe	L	56	−21	−33	−33	9.84
	Posterior cingulate gyrus	R	33	6	−60	12	−7.87
	Anterior cingulate gyrus	R	24	15	36	21	−8.31

## Discussion

This study selected three acupuncture points, *Dazhui* (GV14), *Zhiyang* (GV9), and *Mingmen* (GV4), all belong to the Governor Meridian. The Governor Meridian is closely connected to the brain in Chinese medical theory and is frequently employed in the treatment of neuropsychiatric diseases. In earlier research, our team discovered that stroke patients’ motor dysfunction may be significantly improved by using acupoints from the Governor Meridian (Shi et al., 2015a,b). On this basis, according to many years of clinical experience and other relevant research results, we extracted a group of major acupoint prescription, which have been widely utilized and achieved better clinical outcomes. Unfortunately, there was no significant differences in clinical outcomes in this trial, which may have something to do with the fact that we only included a transitory acupuncture session. In general, the acupuncture therapeutic effects require accumulation from multiple sessions, and once this accumulation reaches a specific point, the curative effects become more noticeable (Xu et al., 2022; Yoon et al., 2022).

The amount of acupuncture stimulation is critical to the therapeutic process. Some foreign studies reported that acupuncture had no extra benefits for stroke rehabilitation (Sze et al., 2002; Leem, 2015). However, Chinese researchers considered that this is because acupuncture does not achieve sufficient stimulation (Shi, 2021; Li B. X. et al., 2022). In this study, we chose the manipulation time of 1-min, 2-min, and 3-min, which was decided according to the findings of the previous study. To better explain the efficacy of manipulation time and exclude the additional effect of the retain needles, we used only one acupuncture session. Interestingly, the volume of the voxel cluster increased with the extension of the manipulation time. Longer periods of acupuncture stimulation have been shown to be more likely to cause changes in brain blood oxygen levels (Li et al., 2006; Napadow et al., 2009). This conclusion is consistent with our findings and suggests that effective acupuncture treatment requires sufficient time for acupuncture stimulation. In the future acupuncture treatment, acupuncturists should pay more attention to the role of manipulation time, and improve the amount of stimulation by increasing the operation time, so as to better play the curative effect.

From the point of dose–response relationship, increasing the manipulation time of acupuncture should theoretically only increase the volume of voxel in the activation brain region. However, our results showed that even with the same prescription, the locations of brain regions activated by different acupuncture manipulation times were not completely consistent. Studies have confirmed that different acupuncture manipulation will cause different neural effects (Shi et al., 2016; Lu et al., 2017). This study shows from a fresh perspective that the manipulation time may also be another key factor in the effective of acupuncture.

According to the ReHo longitudinal study findings, varied acupuncture manipulation times resulted different changes in brain regions. Acupuncture manipulation for 2 min significantly improved the neural activity of the insula. The insula might integrate multiple neural functioning systems, including sensorimotor and cognitive (Primassin et al., 2015; Chen et al., 2019). Insula injury may be the cause of motor disorders in stroke patients, and acupuncture could play a therapeutic role by activating the function of insula (Leigh et al., 2013). A meta-analysis revealed that positive correlation between stroke patients’ insula ReHo levels and motor function scores (Lv et al., 2021). On the other hand, the ReHo values of the activator voxel were at their highest level after 3 min of stimulation. Wu et al. introduced that acupuncture could enhance motor function in stroke patients, and further hypothesized that these improvements might be related to changes in ReHo values of the temporal lobe based on fMRI results (Wu et al., 2017). Here, our findings revealed that neural activity in the insula and the inferior temporal gyrus increased following 2- and 3-min acupuncture manipulation, indicating that these two stimulation ways may be more beneficial in improving motor dysfunction after stroke.

After 1 min of acupuncture manipulation, the neural response of the inferior frontal gyrus was changed. The inferior frontal gyrus is part of the prefrontal cortex, which is widely engaged in higher cognitive activities (Wong et al., 2016; Koyama et al., 2017). After a stroke, individuals might suffer cognitive impairment in addition to motor dysfunction. Previous studies reported that acupuncture can increase glucose metabolism in the frontal lobe and promote the recovery of cognitive function (Huang et al., 2007; Xu et al., 2020). Zhang et al. found that acupuncture also modulated cognitive networks centered on the frontal lobe (Zhang et al., 2021b). Therefore, we believe that one-minute acupuncture stimulation may be more beneficial for stroke patients who also have cognitive impairment. However, this conclusion needs further research to be clear cause the limited sample size.

In this study, our results further found that the changes in blood oxygen levels and voxel cluster volume of the brain activated by 3-min acupuncture stimulation were higher than those activated by shorter stimulation, and the changes mainly occurred in the cingulate gyrus. The cingulate gyrus is a critical component of the default mode network (DMN), which is an important neurophysiological basis for regulating functional activity in the human brain (Borserio et al., 2021). Though the specific function of DMN is unknown, several studies have connected it to a variety of mental disorders such as Alzheimer’s disease, depression and other conditions (Mohan et al., 2016; Harikumar et al., 2021; Hu et al., 2022). Combined with our findings, there is evidence to support that long-term acupuncture stimulation on the Governor Meridian could maintain the *Deqi* sensation and consequently better fulfill the role of *Tiaoshen* (a term from Chinese medicine that is equivalent to regulating emotions, cognition, etc.). Meanwhile, 3-min acupuncture stimulation also activated cerebellar function. The cerebellum dominates sensory movement and has extensive neural fiber connections with the cerebral cortex (Zabihhosseinian et al., 2020; Ashida et al., 2022). Chen et al. further revealed a cortico-subcortical connectivity between the cerebellum and the sensory motor network (SMN) (Chen et al., 2022). Acupuncture can regulate the SMN to relieve hemiplegia, numbness and other symptoms (Wang et al., 2022; Peng et al., 2023). Thus, we consider that 3-min acupuncture stimulation may improve motor dysfunction in stroke patients more effectively by regulating the SMN including the cerebellum compared with shorter stimulation.

Taken together, different acupuncture manipulation times can affect the neural effects of stroke patients, although the ways of influence are different. Previous studies have confirmed that acupuncture could increase functional couplings of DMN and SMN in stroke patients (Ray et al., 2020; Li Y. et al., 2022). Our findings in this study further verified that long-term acupuncture manipulation is more likely to enhance the spontaneous activity of SMN and DMN, which may be the potential neural mechanism of acupuncture manipulation time affecting the recovery of motor dysfunction in stroke patients.

## Limitation

There are two limitations in our trial. (1) Small sample size. Since the trial is a pilot study, the number of subjects recruited is correspondingly small, which may impact the validity of the results. Therefore, our findings need to be further verified. (2) Lack of evaluation on the cumulative effect of acupuncture. Our study pursued the immediate neural effects of acupuncture manipulation, thus there was only one transitory acupuncture session during the whole trial, which also resulted in no significant difference in clinical efficacy outcomes.

## Future research

This pilot study can provide reference for the future research. The association between acupuncture effects and neural function should be better assessed in future studies by utilizing more sensitive clinical efficacy outcomes, such as needle sensation evaluation. Additionally, infarcted tissue may influence the functional activities of the brain (Grosser et al., 2020), and future subgroup analysis can be conducted based on the infarct scope or location to evaluate the robustness of the results. Finally, stroke patients have a variety of symptoms. Studies have pointed out that acupuncture has multiple pathways of action (Wen et al., 2021). Our study also found that the activated brain regions by acupuncture were not limited to the sensorimotor area, the central neural mechanism of acupuncture should be understood more comprehensively from multiple scales in the future.

## Conclusion

Our findings suggest that acupuncture at different manipulation times resulted in different changes of the neural effects in stroke patients, and the volume of activated voxel clusters is positively correlated with the manipulation time. In stroke patients, longer acupuncture manipulation could drive SMN and DMN, which may be the potential neurological mechanism of acupuncture manipulation affecting the recovery of motor dysfunction. Such findings have important implications for comprehending the factors that influence the dose of acupuncture stimulation and provide a more beneficial acupuncture manipulation method for clinical practice.

## Data availability statement

The raw data supporting the conclusions of this article will be made available by the authors, without undue reservation.

## Ethics statement

The studies involving humans were approved by the First Affiliated Hospital of Yunnan University of Chinese Medicine's ethics committee (approved number was 2019-001). The studies were conducted in accordance with the local legislation and institutional requirements. The participants provided their written informed consent to participate in this study.

## Author contributions

YZ: Conceptualization, Data curation, Formal analysis, Writing – original draft, Writing – review & editing. AD: Data curation, Investigation, Writing – original draft. SF: Data curation, Methodology, Writing – original draft. TZ: Investigation, Resources, Writing – original draft. ML: Investigation, Resources, Writing – original draft. JS: Conceptualization, Project administration, Supervision, Writing – original draft, Writing – review & editing. DW: Methodology, Writing – review & editing.

## Footnotes

^1^ http://chictr.org.cn

^2^ http://who.int/icd-10

^3^ http://openstd.samr.gov.cn
